# Psychological predictors of recovery from low back pain: a prospective study

**DOI:** 10.1186/s12891-015-0509-2

**Published:** 2015-03-07

**Authors:** Steven Z George, Jason M Beneciuk

**Affiliations:** Department of Physical Therapy, University of Florida, Gainesville, FL USA; Brooks Rehabilitation, Jacksonville, FL USA; Center for Pain Research and Behavioral Health, University of Florida, Gainesville, FL USA

## Abstract

**Background:**

Recovery from low back pain (LBP) is an important outcome for patients and clinicians. Psychological factors are known to impact the course of LBP but have not been extensively investigated for predicting recovery. The purposes of this study were to: 1) describe LBP recovery rates at 6 months following 4 weeks of physical therapy; 2) identify psychological factors predictive of 6 month recovery status; and 3) identify psychological factors that co-occur with 6 month recovery status.

**Methods:**

This study was a secondary analysis of a prospective cohort of patients (n = 111) receiving outpatient physical therapy for LBP. Patients were administered the STarT Back Screening Tool (SBT), individual psychological measures, a numerical pain rating scale (NPRS) and Roland Morris Disability Questionnaire (RMDQ) at intake, 4-week, and 6-month assessments. LBP recovery was operationally defined based on meeting NPRS = 0/10 and RMDQ ≤ 2 criterion at 6-month follow-up assessment. Recovery groups were then compared for differences on all variables at intake and on individual psychological measures at 6-months. Discriminant function analysis (DFA) identified which descriptive variables were predictive of recovery status.

**Results:**

The 6-month recovery rate was 14/111 (12.6%) for the combined NPRS and RMDQ criterion. Non-recovered patients were associated with SBT risk status (p = 0.004), higher intake pain intensity (p = .008) and higher depressive symptoms (p < .001) scores compared to recovered patients. The overall accuracy for intake classification using DFA was 87.2% with SBT risk status, pain intensity, and depressive symptoms all making unique contributions. At 6-months, non-recovered patients had higher fear-avoidance, kinesiophobia, and depressive symptoms (p’s < .001) compared to recovered patients. The overall accuracy for 6-month classification using DFA was 86.4% with fear-avoidance, kinesiophobia, and depressive symptoms all making unique contributions.

**Conclusions:**

Our findings indicated that psychological risk status, depressive symptoms, and pain intensity were predictive of 6 month recovery status. Furthermore elevated fear-avoidance, kinesiophobia, and depressive symptoms co-occurred with non-recovery at 6 months. Future studies should investigate whether stratified psychologically informed treatment options have the potential to improve recovery rates for those most at risk for non-recovery.

## Background

Outcome assessment for low back pain (LBP) has received considerable attention in the literature with pain intensity, back specific function, general health status, work disability, and patient satisfaction identified as 5 core domains [1]. In addition, there has been effort in determining what amount of change in these domains constitutes a “meaningful”, “important”, or “successful” outcome through distribution or anchor based approaches [2,3]. Distribution based approaches include minimal detectable change (MDC) or standardized response mean (SRM), while anchor based approaches include minimal clinically important difference (MCID) or minimal important change (MIC) [2,3]. The change criteria derived from distribution and anchor based approaches differ in the way they are calculated, but both provide the amount of change necessary for a given measure before a meaningful, important, or successful outcome is attained.

Another assessment option is the use of absolute criterion for determining LBP outcomes. Recovery from LBP is one such absolute criterion that has been advocated for because it is a construct of fundamental importance to the patient and clinician [4,5]. Recovery from LBP is theorized to be a complex, multifactorial construct but has been commonly operationally defined by 2/5 of the core outcome domains – pain intensity and back specific function [5]. A 2011 systematic review [6] highlighted high priority issues to address in future studies of LBP recovery. In this review the authors reported tremendous assessment variability with 66 different measures reported across 82 studies, including 59 measures reported only once. Only 17 studies followed a multifactorial definition of recovery using combined measures (e.g. pain and function) and 1 study assessed combined measures of recovery beyond 4 months of follow up [6]. Furthermore, psychological factors are predictive of individual low back pain outcomes for pain and disability [7], yet their association with composite recovery measures has not been widely explored. Establishing whether psychological factors are also predictive of composite recovery measures for LBP has implications for future clinical and research initiatives since psychological assessment is now commonly recommended by clinical guidelines [8,9].

Therefore, the purpose of this analysis was to investigate low back pain recovery in a cohort of patients receiving physical therapy. This analysis directly addresses limitations identified in the systematic review from Kamper et al. [6] by using a previously described recovery criterion derived from pain (numerical rating scale) and function (Roland Morris Disability Questionnaire). This analysis will also incorporate psychological measures predictive of low back pain outcomes, but that have not been considered as predictors for a composite recovery metric. Our objectives were to: 1) describe recovery rates at 6 months following 4 weeks of physical therapy; 2) identify psychological factors predictive of 6 month recovery status; and 3) identify psychological factors that co-occur with 6 month recovery status. The results of this analysis will contribute to an important area of outcome assessment by providing novel data on LBP recovery when a composite measure was used. Specifically we will identify psychological factors that could be used to predict future recovery status (objective 2) or considered as an additional component of recovery (objective 3).

## Methods

Data for this prospective observational study were collected between December 14, 2009 and February 5, 2012 from four outpatient physical therapy clinics located in Jacksonville, FL and two outpatient physical therapy clinics located in Gainesville, FL. This analysis represents a secondary analysis from this cohort, which was assembled for a primary analysis of comparing one-dimensional and multi-dimensional psychological measures for prediction of non-recovery related clinical outcomes [10].

### Participants

Consecutive patients seeking treatment for LBP were recruited from participating outpatient physical therapy clinics. All patients were referred for physical therapy by a physician and screened for eligibility by a study physical therapist. Potential study participants met both of the following criteria before being enrolled into this study: 1) adults between the ages of 18 and 65 years seeking physical therapy for LBP (defined as having symptoms at T12 or lower, including radiating pain into the buttocks and lower extremity) and 2) the ability to read and speak the English language. These broad inclusion criteria were to allow for a cohort that was representative of routine clinical practice. Potential study participants were ineligible to participate in this study if any of the following criteria were met: 1) the presence of systemic involvement related to metastatic or visceral disease; 2) recent spinal fracture; 3) osteoporosis; or 4) pregnancy. Physical therapists provided all patients that met study eligibility criteria with a brief explanation of the study and a study advertisement with primary investigator contact information. Clinicians emphasized to patients that participating in this study would not dictate the treatment they received for their LBP and if they elected not to participate they would receive the same treatment. All participants provided written informed consent using official documents that were in compliance with and approved by the University of Florida Institutional Review Board.

### Procedures

Eligible and consenting participants were assessed at intake, after 4 weeks of treatment, and then 6 months later. Data collection for demographic, clinical, psychological, and outcome factors was through self-report. Clinical examination data was collected for physical impairment. Participants were included in this analysis if they had completed follow up data at 6 months, while participants with missing data were excluded.

### Predictive measures

Psychological factors were the primary focus of this analysis, but demographic, clinical, pain intensity, and disability measures were also included in the models. The rationale for including these competing predictive variables was to investigate their predictive potential for recovery, and to provide appropriate statistical control in multivariate models**.** The specific measurement categories are described in more detail below.

#### Demographic and clinical

Study participants completed a standardized self-report questionnaire consisting of questions related to age, sex, prior LBP episodes, and current symptom duration. Responses to prior LBP episodes over the previous 12-months were categorized as “yes” or “no”. Symptom duration for LBP was categorized into acute (≤14 days), subacute (15 – 90 days), and chronic (≥90 days).

#### Pain intensity and disability

Pain intensity was measured with a numerical pain rating scale (NPRS) ranging from “0” (no pain) to “10” (worst pain imaginable) [11-13]. Participants were asked to rate their current pain intensity, as well as their best and worst level of pain intensity over the past 24 hours. These three pain ratings were averaged and used as the NPRS variable in this study [14]. The Roland-Morris Disability Questionnaire RMDQ was used to measure LBP disability. The RMDQ has 24 items that assesses the functional status over the past 24 hours in patients with LBP [15]. The RMDQ has a range of 0 (no disability due to LBP) to 24 (maximum disability due to LBP), with higher scores indicating higher disability from LBP. In previous studies the RMDQ has been found to have high levels of test-retest reliability, internal consistency, validity, and responsiveness [15,16].

#### Psychological – screening tool

The STarT Back Tool (SBT) is a screening measure consisting of 9-items related to physical and psychosocial factors used to categorize patients with LBP in primary care settings based on their risk for poor future disability outcomes [17]. The rationale for including a psychological screening tool was that it would provide an analytical option that may allow for efficient prediction of recovery status. Based on overall and psychosocial subscale scoring, the SBT categorizes patients as ‘high-risk’ (psychosocial subscale scores ≥4) in which high levels of psychosocial prognostic factors are present with or without physical factors present, ‘medium-risk’ (overall score >3; psychosocial subscale score <4) in which physical and psychosocial factors are present, but not a high level of psychosocial factors, or ‘low-risk’ (overall score 0–3) in which few prognostic factors are present [18].

#### Psychological – individual measures

In addition to the SBT, data on individual psychological constructs was collected. The rationale for including individual measures was to provide more sensitive consideration of individual psychological constructs when predicting recovery status. Fear-avoidance beliefs specific to LBP were assessed with the Fear-Avoidance Beliefs Questionnaire (FABQ) [19]. The FABQ consists of a 4-item FABQ physical activity scale (FABQ-PA, potentially ranging from 0 to 24) and a 7-item FABQ work scale (FABQ-W, potentially ranging from 0 to 42), with higher scores indicating higher levels of fear-avoidance beliefs for both FABQ scales. The PCS was used to assess the degree of catastrophic cognitions due to LBP [20]. Pain catastrophizing has been broadly defined as an exaggerated negative orientation towards actual or anticipated pain experiences [20]. The Pain Catastrophizing Scale (PCS) is a 13-item questionnaire with a potential range of 0 to 52, with higher scores indicating higher levels of pain catastrophizing. The Tampa Scale of Kinesiophobia (TSK) was used to assess the degree of fear of movement and injury or re-injury in individuals with LBP [21]. The TSK-11 is an 11-item questionnaire with a potential range of 11 to 44, with higher scores indicating greater fear of movement and injury or re-injury due to pain. The Patient Health Questionnaire (PHQ) was used to assess the degree to which depressive symptoms have on patients with LBP [22]. The PHQ-9 is a 9-item questionnaire with a potential range of 0 to 27, with higher scores indicating elevated depressive symptoms. The FABQ-PA, FABQ-W, and TSK-11 each assess different aspects of pain-related fear. These measures were included as individual predictors because we expected positive correlation between the measures, but not enough to indicate construct redundancy or collinearity (r’s range from 0.28 to 0.62) [23,24]. Therefore each questionnaire was included in the models to provide a preliminary indication of which specific aspect of pain-related fear was predictive of LBP recovery.

### Outcome measure

#### Recovery

Recovery was determined at 6-months with a combination of the aforementioned NPRS and RMDQ. The definition of recovery used in this study was for an NPRS score of 0 and a RMDQ score of ≤ 2 because individually these criterion accurately predicted patients rating themselves as completely recovered in a previous study [5].

### Data analysis

All data analyses were performed using SPSS, Version 21.0. Descriptive statistics were first calculated for all intake descriptive and outcome variables. Then to address our objectives the following analyses were performed: 1) 6-month recovery rates were determined from the percentage of those that met the recovery criterion (“recovered”) compared to those that did not meet the criterion (“not recovered”); 2) The recovered and not recovered groups were compared for intake differences on all variables using independent t-tests (for continuous measures) and chi-square tests (for categorical measures); 3) The recovered and not recovered groups were further compared for differences in 6-month scores on only the psychological measures by independent t-tests. If multiple statistical differences existed for analysis in steps 2 or 3 then discriminant function analysis (DFA) run with simultaneous entry method was used to identify which descriptive variables were associated with recovery in multivariate models. Briefly, DFA is a multivariate statistical procedure used to determine if a set of variables can predict group membership (i.e. recovery status for this analysis) [25]. Both models were used to discriminate recovery groups, but one DFA model included predictive variables from intake measures (Purpose 2) and a separate DFA model included concurrent psychological variables at 6 months (Purpose 3). Canonical correlations were reported as a measure of the relationship between those “recovered” and “not recovered” and the discriminant function. A summary of classification results from the DFA was generated using jack-knifed procedures (i.e., one case at a time deleted) to evaluate for accuracy in LBP recovery group categorization. All analyses were performed at an alpha level of 0.01 to account for the number of variables in the analyses. This level was selected because it partially corrects for multiple correction while not being overly conservative in preventing potentially clinically relevant factors to be considered as predictors of recovery.

## Results

These analyses included data from 111/146 participants with completed NPRS and RMDQ data at 6 months [10]. Participants (mean ± sd_ that completed the 6-month follow-up assessment were older (43.5 ± 12.3 years) than non-completers (34.6 ± 14.8 years) (p < .05). No other differences between completers and non-completers were observed. Baseline demographic data for the entire sample and recovery groups is presented in Table 1. Visual inspection indicated that normal distribution for the NPRS, RMDQ and all individual psychological measures was approximated.

**Table 1:** **Intake differences for recovery groups**

**Variable**	**Total sample (n = 111)**	**Recovered (n = 14)**	**Not Recovered (n = 97)**	**p-value** ^†^
Age (years)	43.5 ± 12.4 (45.0)	40.2 ± 14.1 (45.0)	43.8 ± 12.1 (46.0)	.290
Sex (n, female)	72 (64.9%)	7/72 (9.7%)	65/72 (90.3%)	.213
**Prior** LBP episodes (n, yes)	70 (63.1%)	7/70 (10.0%)	63/70 (90.0%)	.279
Symptom duration (n, chronic)	53 (47.7%)	6/53 (11.3%)	47/53 (88.7%)	.618
NPRS	5.4 ± 1.9 (5.3)	4.1 ± 2.2 (4.3)	5.6 ± 1.8 (5.7)	.008^*^
RMDQ	11.1 ± 6.0 (10.0)	7.7 ± 5.4 (7.0)	11.6 ± 6.0 (11.0)	.022
SBT status				
Low risk	38 (34.2%)	10/38 (26.3%)	28/38 (73.7%)	.004^*^
Medium risk	45 (40.5%)	4/45 (8.9%)	41/45 (91.1%)	
High risk	28 (25.2%)	0/28 (0.0%)	28/28 (100.0%)	
FABQ-PA	14.4 ± 6.1 (14.0)	12.7 ± 7.5 (13.0)	14.7 ± 5.9 (15.0)	.267
FABQ-W	12.6 ± 10.9 (11.0)	6.6 ± 7.1 (5.0)	13.7 ± 11.1 (11.0)	.025
PCS	16.4 ± 12.0 (14.0)	13.8 ± 13.1 (11.0)	16.9 ± 11.9 (15.0)	.385
TSK-11	25.2 ± 6.9 (25.0)	23.2 ± 8.0 (21.0)	25.7 ± 6.7 (25.0)	.251
PHQ-9	7.5 ± 6.0 (6.0)	2.3 ± 2.8 (2.0)	8.2 ± 6.1 (7.0)	<.001^*^

All values for continuous measures are reported as mean ± standard deviation and (median) estimates. All values for categorical measures are reported as frequency count (percentage) estimates in relation to respective category of total study sample. (†) indicates significance level for recovery status comparisons using independent samples t-tests (for continuous measures) and chi-square tests (for categorical measures); an alpha level of 0.01 was used for all analyses. (*) indicates p < 0.01.

*Abbreviations:* *LBP* low back pain, *NPRS* Numerical Pain Rating Scale, *RMDQ* Roland-Morris Disability Questionnaire, *SBT* STarT Back Tool, *FABQ-PA* Fear-Avoidance Beliefs Questionnaire (physical activity scale), *FABQ-W* Fear-Avoidance Beliefs Questionnaire (work scale), *PCS* Pain Catastrophizing Scale, *TSK-11* Tampa Scale for Kinesiophobia (11-item version), *PHQ-9* Patient Health Questionnaire (9-item version).

### Recovery rate

The 6 month recovery rate was 14/111 (12.6%) for the combined NPRS = 0 and RMDQ ≤ 2 criterion. Rates for meeting the individual criteria were 14/111 (12.6%) for the NPRS and 36/111 (32.4%) for the RMDQ.

### Intake differences

The intake differences for recovery groups are presented in Table 1. High risk SBT status had the lowest recovery rates. Other intake differences included NPRS and PHQ-9 scores, which were higher scores for those not recovered. None of the other intake variables differed based on 6 month recovery status (Table 1). Multivariate analysis findings via DFA indicated that intake SBT status (Wilks’ λ = .90, p < .001), NPRS (Wilks’ λ = .94, p = .010) and PHQ-9 (Wilks’ λ = .90, p < .001) scores each contributed uniquely to LBP recovery. The overall test of the single discriminant function was significant (*χ*2 (3) = 15.96, Wilks’ λ = .86, p < .001) indicating that predictor scores discriminated between the two LBP recovery groups accounting for 14.4% (canonical R = .38) of the total variance. Inspection of standardized canonical discriminant function coefficients (analogous to multiple regression beta weights) indicated that intake SBT status (.491) and PHQ-9 scores (.512) demonstrated moderate positive relationships with the discriminant function for recovery, whereas intake NPRS scores (.259) demonstrated a weaker positive relationship. The overall accuracy for classification using the discriminant function for recovery status was 87.2% using the cross-validated jackknifing technique.

### 6-month differences in individual psychological measures

Six month differences in psychological measures for the recovery groups is presented in Table 2**.** Differences were noted for the FABQ-PA, TSK-11, and the PHQ-9 (higher scores for those not recovered). Multivariate analysis findings via DFA suggested that 6 month FABQ-PA (Wilks’ λ = .88, p < .001). TSK-11 (Wilks’ λ = .80, p < .001) and PHQ-9 (Wilks’ λ = .89, p < .001) scores each contributed uniquely to LBP recovery categorization. The overall test of the single discriminant function was significant (*χ*2 (3) = 24.96, Wilks’ λ = .79, p < .001) indicating that scores discriminated between the two LBP recovery groups accounting for 21.2% (canonical R = .46) of the total variance. Inspection of standardized canonical discriminant function coefficients indicated that 6-month TSK-11 scores (.707) demonstrated a strong positive relationship with the discriminant function for recovery, whereas 6-month FABQ-PA (.237) and PHQ-9 (.209) scores demonstrated a weaker positive relationship. The overall accuracy for classification using the discriminant function for recovery status was 86.4% using the cross-validated jackknifing technique.

**Table 2:** **Six-month differences for recovery groups**

**Psychological Measure**	**Recovered (n = 14)**	**Not Recovered (n = 97)**	**p-value** ^†^
FABQ-PA	5.1 ± 5.7 (3.0)	12.5 ± 6.7 (13.0)	<.001^*^
FABQ-W	4.2 ± 5.6 (2.0)	12.5 ± 13.1 (9.0)	.022
PCS	6.1 ± 10.1 (0.5)	11.6 ± 11.5 (8.0)	.097
TSK-11	13.8 ± 4.8 (11.0)	24.0 ± 7.1 (23.0)	<.001^*^
PHQ-9	0.1 ± 0.3 (0.0)	4.8 ± 4.8 (3.0)	<.001^*^

All values are reported as mean ± standard deviation and (median) estimates. (†) indicates significance level for recovery status comparisons using independent samples t-tests; an alpha level of 0.01 was used for all analyses. (*) indicates p < 0.01.

*Abbreviations:* *FABQ-PA* Fear-Avoidance Beliefs Questionnaire (physical activity scale), *FABQ-W* Fear-Avoidance Beliefs Questionnaire (work scale), *PCS* Pain Catastrophizing Scale, *TSK-11* Tampa Scale for Kinesiophobia (11-item version), *PHQ-9* Patient Health Questionnaire (9-item version).

## Discussion

This analysis investigated 6 month recovery for a cohort of patients receiving outpatient physical therapy. An unexpected finding was that the recovery rate from this cohort was low (12.6%), but consistent with a previous report that pooled four clinical studies of patients receiving various treatments for LBP [5]. In this cohort the low recovery rate was completely driven by the lack of patients meeting the pain intensity criterion (ie, NPRS = 0), which is consistent with earlier reports that looked at the individual components of this particular composite recovery definition [5]. More patients met the individual criterion for the RMDQ (32.4%) showing how a composite measure adds more stringency to sole reliance on disability measures. Available studies indicate consistency with the pain intensity criterion being the rate limiting factor for recovery, but more research is needed in this area to further clarify what the expected recovery rates and outcomes are following a trial of outpatient physical therapy for LBP.

At intake, high risk status on a multidimensional psychological screening tool, higher depressive symptoms, and higher pain intensity were uniquely predictive of not recovering at 6 months. At 6 months, higher depressive symptoms, fear-avoidance related to physical activity, and kinesiophobia scores were uniquely associated with non-recovery at 6 months. Collectively these findings add to the existing literature by identifying psychological factors 1) predictive of a composite recovery measure and 2) that co-occur with non-recovery at 6 months.

It was not an unexpected finding that SBT high risk status, depressive symptoms, and pain intensity, were predictive of recovery status. These variables had been identified in previous studies as predictors for individual outcome measures [10,26,27], but they had not been investigated for their potential to predict a composite recovery measure to the best of our knowledge. The importance of psychological factors link to recovery comes from clinical guideline recommendations for routine psychological or psychosocial assessment of patients with LBP [8,9]. Findings from the current study provide convergent evidence that intake factors should be used as clinical predictors for pain and disability outcomes. Risk status on the SBT, depressive symptoms, and pain intensity appear to be robust predictors with good potential for individual outcome measures and a composite recovery measure.

In particular this analysis provided additional support for the utility of the SBT as it clearly discriminated recovery status in this cohort. The finding that 26% of the low risk, 9% of the medium risk, and 0% of the high risk patients met a stringent recovery criterion is noteworthy. Future studies are needed to determine if providing stratified treatment that incorporates psychologically informed interventions [28] improves these rates for medium and high risk patients. To date, trials using the SBT have incorporated the RMDQ as a primary outcome measure and reported small to moderate improvements for high risk patients that receive stratified care [29,30]. The impact this RMDQ improvement has on recovery rates for high risk patients is unknown and merits future research given that it is often the change in pain intensity that limits recovery [5]. If patients identified as medium or high risk by the SBT are less likely to recover from low back pain even with psychologically informed approaches then other treatment methods need to be investigated to determine what approaches are necessary to provide higher recovery rates in these risk subgroups. Therefore, future clinical trials for low back pain should consider incorporating recovery measures to determine if the benefits of a particular approach are enough to alter recovery rates, as information on the recovery outcome may be more meaningful for patients and policy makers.

The elevated psychological scores for those not recovered at 6 months demonstrate the impact of continued low back pain after seeking healthcare. Differences in psychological factors were minimal at intake (depressive symptoms only), but included fear-avoidance and kinesiophobia at 6 months. Interestingly, there were no differences in pain catastrophizing at intake or at 6 months based on recovery status. These findings have theoretical implications for those interested in a fear-avoidance model of musculoskeletal pain [31,32] by suggesting that elevation in fear related constructs may have a larger influence on recovery in comparison to pain catastrophizing. These findings also have practical implications for those interested in expanding composite recovery criterion beyond pain and disability to include a psychological element. Previous studies have indicated improvements in psychological measures to be positively associated with beneficial clinical outcomes [33-35]. Our results suggest that a psychological element of recovery should include a measure of fear-avoidance or kinesiophobia. These constructs seem more appropriate to add to the recovery criterion in comparison to depressive symptoms because differences in fear related factors were not apparent at intake, while elevated depressive symptoms were. A reasonable clinical approach that could be advocated from this study is that patients identified as medium or high risk by SBT at baseline should be monitored for treatment response by the FABQ or TSK, with increases in these scores being an additional indication of non-recovery.

This study also has several limitations to consider when interpreting these results. First, this was a secondary analysis of this cohort, as the original intent was to compare relative predictive ability of psychological measures for individual outcomes. Second, the physical therapy interventions used to treat patients in this cohort were not standardized or recorded. Therefore, we cannot make any inferences on whether any particular treatment approaches implemented within physical therapy had an influence on recovery rates. Third, the use of self-report outcome measures provided a single perspective on 6 month recovery. Future studies of recovery should incorporate physical impairment or performance based measures to compliment the more commonly used self-report measures used in low back pain studies. Fourth, the variance accounted for by the DFA was only 14.4%, suggesting additional factors are involved with prediction of recovery. Future studies should consider incorporating other relevant factors not included in these models, including psychological (e.g. expectation of recovery) and non-psychological (e.g. genetic risk and pain sensitivity) predictors. Finally, the 11-point Global Back Recovery Scale, for a simple measure of global recovery, and the Patient-Generated Index of Quality of Life–Back Pain, to evaluate specific relevant dimensions of recovery have both been recommended as an adjunct to the existing corset of LBP outcome measures [36]. Therefore, future studies should incorporate these measures as an indicator for LBP recovery.

## Conclusion

Composite measures of low back pain recovery based on absolute criterion provide important information on clinical outcome. However in the past these measures have not been widely reported, and when they are reported have not been done in a standard manner. This analysis investigated psychological factors for their association with a previously described composite measure for recovery consisting of the NPRS for pain intensity and the RMDQ for disability. Our findings indicated that psychological risk status, depressive symptoms, and pain intensity were predictive of 6 month recovery status and elevated fear-avoidance, kinesiophobia, and depressive symptoms co-occurred with non-recovery at 6 months. Future studies should investigate whether available stratified treatment options have the potential to improve recovery rates for those most at risk for not recovering.

## Abbreviations

LBP: Low back pain
SBT: STarT Back Screening Tool
NPRS: Numerical pain rating scale
RMDQ: Roland Morris Disability Questionnaire
DFA: Discriminant function analysis
FABQ-PA: Fear Avoidance Beliefs Questionnaire Physical Activity Scale
FABQ-W: Fear Avoidance Beliefs Questionnaire Work Scale
TSK: Tampa Scale of Kinesiophobia
PCS: Pain Catastrophizing Scale
PHQ: Patient Health Questionnaire
